# Effect of Ornithine α-Ketoglutarate on Intestinal Microbiota and Serum Inflammatory Cytokines in Dextran Sulfate Sodium Induced Colitis

**DOI:** 10.3390/nu15112476

**Published:** 2023-05-26

**Authors:** Tao Wang, Junquan Tian, Wenxuan Su, Fan Yang, Jie Yin, Qian Jiang, Yuying Li, Kang Yao, Tiejun Li, Yulong Yin

**Affiliations:** 1 Laboratory of Animal Nutritional Physiology and Metabolic Process, Key Laboratory of Agro-Ecological Processes in Subtropical Region, National Engineering Laboratory for Pollution Control and Waste Utilization in Livestock and Poultry Production, Institute of Subtropical Agriculture, Chinese Academy of Sciences, Changsha 410125, China; wttrue@163.com (T.W.);; 2 University of Chinese Academy of Sciences, Beijing 100008, China; 3 College of Animal Science and Technology, Hunan Agricultural University, Changsha 410125, China; 4 Institute of Bast Fiber Crops, Chinese Academy of Agricultural Sciences, Changsha 410205, China

**Keywords:** ornithine α-ketoglutarate, dextran sulfate sodium, colitis, intestinal microbiota, inflammatory cytokines

## Abstract

Ornithine α-ketoglutarate (OKG), a nutritional compound, is an amino acid salt with anti-oxidative and anti-inflammatory effects on humans and animals. Ulcerative colitis (UC), as an inflammatory bowel disease (IBD), leads to chronic intestinal inflammatory dysfunction. This study evaluated the optimal dosage of OKG in healthy mice. Then, a mouse model of acute colitis was established using dextran sodium sulfate (DSS), and the preventive effect of OKG on DSS-induced colitis in mice was explored through analysis of serum inflammatory cytokines and fecal microbiota. Initially, the mice were randomly divided into a control group, a group given a low dose of OKG (LOKG: 0.5%), a group given a medium dose of OKG (MOKG: 1%), and a group given a high dose of OKG (HOKG: 1.5%); they remained in these groups for the entire 14-day experimental period. Our results demonstrated that 1% OKG supplementation increased body weight, serum growth hormone (GH), insulin (INS), alkaline phosphatase (ALP), Tyr, and His and decreased urea nitrogen (BUN), NH_3_L, and Ile. Then, a 2 × 2 factor design was used for a total of 40 mice, with diet (a standard diet or a 1% OKG diet) and challenge (4% DSS or not) as the main factors. During days 14 to 21, the DSS mice were administered 4% DSS to induce colitis. The results revealed that OKG alleviated weight loss and reversed the increases in colonic histological damage induced by DSS. OKG also increased serum IL-10 secretion. Moreover, OKG enhanced the abundance of Firmicutes and decreased that of Bacteriodetes at the phylum level and particularly enhanced the abundance of Alistipes and reduced that of Parabacterioides at the genus level. Our results indicated that OKG promotes growth performance and hormone secretion and regulates serum biochemical indicators and amino acid concentrations. Furthermore, 1% OKG supplementation prevents DSS-induced colitis in mice via altering microbial compositions and reducing the secretion of inflammatory cytokines in serum.

## 1. Introduction

Ulcerative colitis (UC) is a chronic inflammatory bowel disease (IBD) characterized by symptoms such as decreased appetite, weight loss, abdominal pain, diarrhea, and bloody stool. The etiology and pathogenesis of UC are not yet clear, but the disease may be associated with genetic susceptibility, intestinal microbiota, and/or environmental or immune factors [1]. UC is characterized by inflammation of the colon and rectum mucosa as seen through pathological and superficial inflammation screening in histology [2]. Currently, the incidence and prevalence of IBD are expanding worldwide, especially in developing countries such as China. In addition, studies have shown that acute DSS-induced colitis promotes intestinal microbial dysregulation in mice, including reducing bacterial species richness and altering bacterial community compositions [3,4]. Chirlaque et al. reported that intestinal bacteria are essential for the development of common DSS-induced colitis [5]. The loss of a microbiome significantly reduces the inflammatory response of the large intestine after DSS exposure and impairs intestinal barrier function. The current medications used to treat UC involve aminosalicylates, glucocorticoids, corticosteroids, and immunosuppressants [6,7]. However, these drugs frequently only alleviate symptoms instead of curing disease and may induce substantial side effects. Therefore, the study of preventive drugs for UC is a current research hotspot, and nutritional regulation is one of the safest and most effective ways to prevent IBD.

OKG is an amino acid complex salt formed by binding ornithine and α-ketoglutaric acid with an ionic bond. It provides multiple physiological benefits, including promoting growth [8], antioxidant activity [9,10], anticancer effects [11], anti-inflammatory properties, and decreasing tissue damage [12]. Additionally, amino acid complex salts not only possess the functions of two amino acids but also mutually enhance their physiological activities in the body [13]. Ornithine enters the urea cycle in the body, serving as a precursor substance for arginine, citrulline, and proline and a direct precursor for the synthesis of polyamines (spermidine, spermine, and putrescine), which can promote growth hormone secretion and animal growth during the juvenile period. Polyamines are a momentary source of energy for cells and can alleviate intestinal damage and reduce inflammatory responses, thus relieving weaning stress [14,15]. α-Ketoglutaric acid is the central molecule of the tricarboxylic acid cycle, serving as a precursor substance for glutamate and glutamine and an important energy source for intestinal cells, playing a major role in protecting the intestinal mucosal barrier [16].

Our previous studies also indicated that the supplementation of 0.5% or 1% OKG alters gut microbes, raises serum amino acids levels, and alleviates growth inhibition in chronic oxidative stress-induced and enterotoxigenic Escherichia coli-infected pigs [17,18]. A dosage of 0.2% or 0.4% OKG can increase body weight gain and enhance pectoral muscle development of chicks [8]. Dietary supplementation with 0.75% OKG significantly improves the growth performance of lactating piglets [19]. OKG provides various benefits in terms of inflammatory responses and growth performance, but its anti-inflammatory mechanism in IBD is still largely obscure. Therefore, we aimed to explore the potential mechanism by which OKG protects against DSS-induced colitis.

## 2. Materials and Methods

### 2.1. Animals

The murine experiments were approved by the Animal Care and Use Committee of the Institute of Subtropical Agriculture, Chinese Academy of Science (no. ISA-2018-4-25). Female ICR mice were purchased from SLAC Laboratory Animal Central (Changsha, China). All mice were allowed free access to food and drinking water and were maintained under standard environment during the experiments. The basal diet was based on Research Diets [20], and the three OKG diets contained 0.5%, 1%, and 1.5% OKG, respectively.

### 2.2. OKG Treatment

ICR mice (24.23 ± 0.96 g, 6 weeks) were randomly divided into four groups (*n* = 10 each), which were designated as the control (basal diet), LOKG (low dose of OKG, 0.5% OKG), MOKG (medium dose of OKG, 1% OKG), and HOKG (high dose of OKG, 1.5% OKG) groups (*n* = 10). Body weights of the mice were monitored daily. Blood samples were collected from the retro-orbital sinus at the end of the experiment.

### 2.3. OKG Treatment in DSS-Induced Colitis

Forty mice (25.98 ± 0.78 g) were randomly assigned to receive either a basal diet (CON group) or a diet containing OKG (*n* = 20). Each group was further divided into two subgroups randomly. The appropriate dosage of OKG was selected based on the above experimental results. Our study lasted 21 days. From days 14 to 21, the DSS-treated mice received 4 % DSS (w v^−1^, molecular mass of 36,000–50,000 Da; MP Biomedicals, Solon, OH, USA) in their drinking water to induce colonic inflammation. The treatment regimens for each group of mice are shown in Figure 2A. During the feeding process, the body weights of the mice were recorded daily. In the last 7 days, fecal shape and blood in stool were observed daily. At the end of the experiment, blood was collected from the retro-orbital sinus. Then, the mice were humanely euthanized. Thereafter, the colon lengths were measured, the middle part of the colon was stored in 4% formaldehyde for histological analysis, and fecal samples were collected for microbiota analyses.

### 2.4. Serum Biochemical Parameters

To measure serum biochemical parameters—including total protein (TP, 03183734190), albumin (ALB, 03183688122), urea nitrogen (BUN, 04460715190), uric acid (UA, 03183807190), glucose (GLU, 04404483190), NH_3_L (20766682322), alanine aminotransferase (ALT, 20764957322), aspartate aminotransferase (AST, 20764949322), and alkaline phosphatase (ALP, 803333701190) levels—in mice, Cobas c-311 Coulter chemistry analyzer (Roche, Shanghai, China) was used [21].

### 2.5. Amino Acids

Serum amino acids (Lys, Met, Thr, Trp, Glu, Asp, Val, Ile, Leu, Phe, Arg, Ser, His, Gly, Ala, Pro, Cys, and Tyr) were analyzed using a 1260 liquid chromatography (Agilent 1260) [22,23].

### 2.6. Clinical Disease Activity Index

Mouse stools were scored on day 21 to determine the disease activity index (DAI). Fecal consistency was scored as 0—firm, 1—slightly soft, 2—very soft, and 3—watery and soft (diarrhea). Fecal blood was scored as 0—normal color, 1—brownish color, 2—reddish color, and 3—bloody red [24].

### 2.7. Colonic HE Staining

Colon samples were fixed with 4% formalin and embedded in paraffin. Then, the 8 μm thick slices were stained with H&E and viewed using Caseviewer software 2.3 [25]. According to the classification of inflammation severity described previously [26] (Table 1), the colon tissue samples were evaluated and classified.

### 2.8. Inflammatory Cytokines in Serum

An ELISA assay kit, which was employed as per manufacturer’s instructions (Jiangsu Yutong Biological Technology Co., Ltd., Yancheng, China), was used to detect tumor necrosis factor-α (TNF-α), interferon-γ (IFN-γ), interleukin–1β (IL-1β), IL-10, growth hormone (GH), and insulin (INS) levels in serum.

### 2.9. Bacterial Profiling

Genomic DNA was extracted from fecal samples (*n* = 6, 3 n/cage), and the DNA concentration and purity were determined using a 1% agarose gel [27,28]. According to the concentration, DNA in the solution was diluted to 1 ng μL-1 with sterile water [20]. Specific primers (16S V3 + V4) with barcodes were used to amplify bacterial 16s rRNA gene. Then, sequencing libraries were generated, assessed, and sequenced using an Illumina MiSeq Sequencer [20]. The original tags were paired, filtered, and analyzed for operational taxonomic unit (OTU) clusters [17,29]. Observed species; Shannon, Simpson, and Chao1 indices; Abundance-based Coverage Estimator (ACE); goods coverage; and phylogenetic diversity (PD) were measured to determine α-diversity. Furthermore, the relative abundances of the four groups at the phylum and genus levels were compared, and the top 10 most-abundant families were defined as dominant genera flora and compared. Microbial functions were predicted using PICRUSt based on KEGG pathways. Raw sequences are available in the NCBI SRA database and have been assigned the following accession number: PRJNA714735.

### 2.10. Statistical Analysis

All data were subjected to one-way ANOVA or two-way ANOVA using the Tukey test (IBM SPSS statistics 20 software). The statistical model included the effects of induction (saline or DSS), diet (basal or OKG), and their interaction. Data are presented as mean ± SEM. Differences of *p* < 0.05 were considered significant.

## 3. Results

### 3.1. OKG Treatment Improves Growth Performance in Healthy Mice

**Figure 1:**
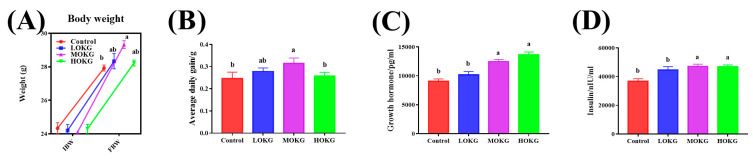
Effects of OKG on growth performance of healthy mice. (**A**) body weight (IBW: initial body weight; FBW: final body weight), (**B**) average daily weight gain, and (**C**,**D**) serum concentrations of growth hormone and insulin. Values within a row with different superscripts (the symbols “a, b”) differ significantly (*p* < 0.05).

As shown in Figure 1, we first measured the body weight and the serum concentrations of growth hormone and insulin in the healthy mice. The MOKG group exhibited a significant increase in both body weight and average daily weight gain (Figure 1A,B). the LOKG or HOKG treatments increased body weight and average daily gain but had no significant effect. In addition, the MOKG and HOKG groups had markedly increased levels of serum growth hormone and insulin (Figure 1C,D). In addition, the dose–response relationship between OKG and insulin secretion in mice was linear.

We further measured the levels of serum biochemical parameters (Table 2) and amino acids (Table 3). The analysis revealed that the LOKG group had significantly decreased serum BUN, Ile, and Tyr levels but increased levels of ALP. The MOKG group presented decreased serum BUN, NH_3_L, and Ile levels but increased levels of GLU, ALP, Tyr, and Phe. Meanwhile, the HOKG group had markedly increased levels of UA, GLU, NH_3_L, Lys, Phe, and His but decreased levels of ALT and AST.

### 3.2. OKG Ameliorates the Body Weight, Colon Length, and DAI of DSS-Induced Colitis in Mice

**Figure 2:**
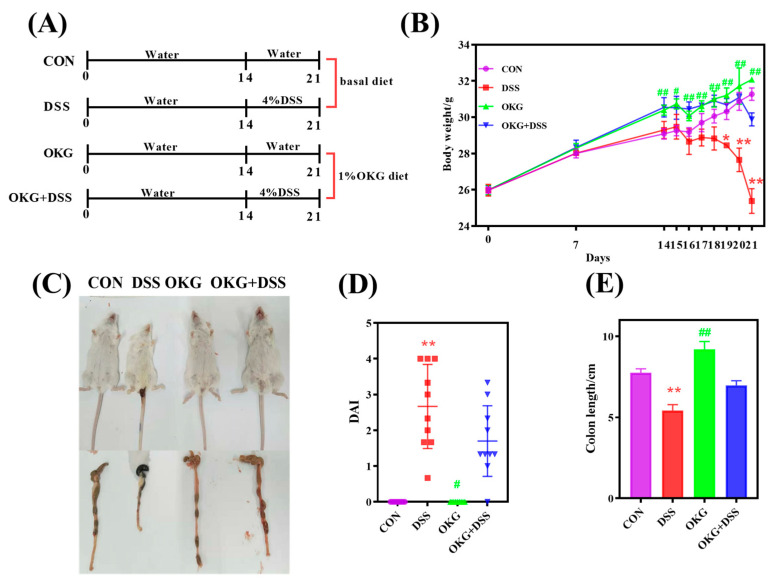
Effects of OKG on body weight, DAI, and colon length of mice with DSS-induced colitis. (**A**) The experimental design; (**B**) body weight. (**C**–**E**) disease activity index (DAI) and colon length. * *p* < 0.05 and ** *p* < 0.01 indicate a statistically significant difference for the challenge (water or DSS). ^#^ *p* < 0.05 and ^##^ *p* < 0.01 indicate a statistically significant difference for the dietary treatment (basal or OKG).

In this experiment, a 4% DSS-induced acute colitis mouse model was used. As shown in Figure 2, the mice given DSS had significantly lower body weights, shorter colon lengths, and higher DAI scores compared with those of the CON group, indicating the successful construction of the model (Figure 2). However, after OKG treatment, the decline in body weight was alleviated, the colon lengths increased significantly, and the DAI scores decreased significantly. Moreover, we observed that the body weights and colon lengths of the OKG mice were higher than those of the control mice, indicating that OKG can prevent colonic damage caused by DSS (to some extent).

### 3.3. OKG Alleviates the Pathological Changes in DSS-Induced Colitis

**Figure 3:**
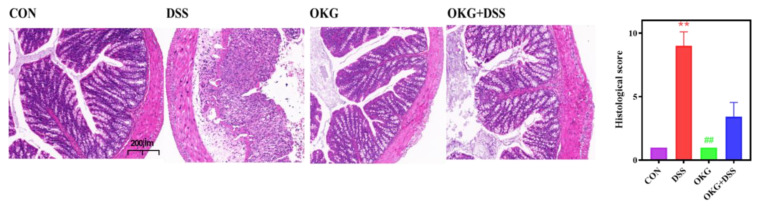
Effects of OKG on the histological scores (**right**) and histological sections (**left**) of mice with DSS-induced colitis. ** *p* < 0.01 indicate a statistically significant difference for challenge (water or DSS). ^##^ *p* < 0.01 indicate a statistically significant difference for dietary treatment (basal or OKG).

As represented in Figure 3, compared with the normal tissue sections (CON and OKG group), the colons of the DSS group exhibited the typical pathological features of colitis, including erosive lesions, and were infiltrated by inflammatory cells. The histological score of a colon partially reflects its health status. In the OKG+DSS group, although there were some inflammatory lesions, the severity of the histological injuries and inflammation was lower than that measured in the DSS mice.

### 3.4. OKG Affects the Inflammatory Cytokines in DSS-Induced Colitis

**Figure 4:**
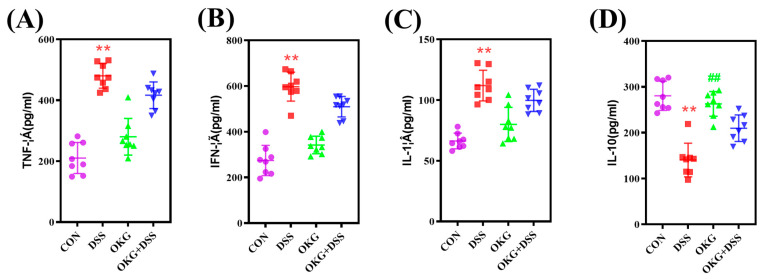
Effects of OKG on the serum TNF-α (**A**), IFN-γ (**B**), IL-1β (**C**), and IL-10 (**D**) levels of mice with DSS-induced colitis. ** *p* < 0.01 indicate a statistically significant difference for challenge (water or DSS). ^##^ *p* < 0.01 indicate a statistically significant difference for dietary treatment (basal or OKG).

Compared with the CON group, the DSS group exhibited a significant increase in serum TNF-α, IFN-γ, and IL-1β levels but decreased IL-10 secretion (Figure 4). Serum, IL-10 was significantly up-regulated in the mice administered the OKG treatment. Compared with the DSS group, the DSS+OKG group did not exhibit affected serum TNF-α, IFN-γ, IL-1β, or IL-10 levels.

### 3.5. OKG Affects the Gut Microbiota of Subjects with DSS-Induced Colitis

**Figure 5:**
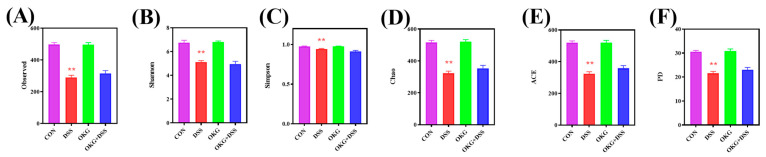
Effects of OKG on the observed index (**A**), Shannon index (**B**), Simpson index (**C**), Chao index (**D**), ACE index (**E**), and PD index (**F**) of mice with DSS-induced colitis. ** *p* < 0.01 indicate a statistically significant difference for challenge (water or DSS).

**Figure 6:**
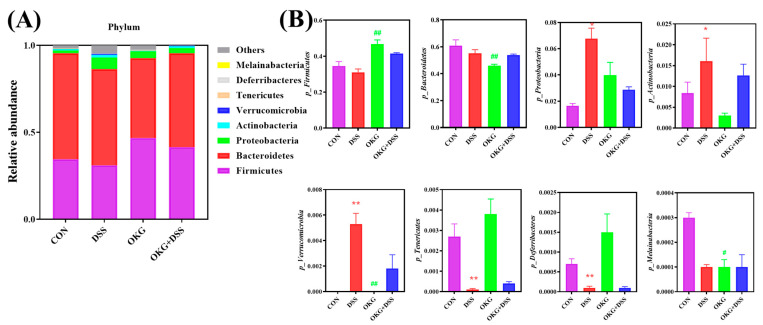
Effects of OKG on the relative abundance of microbiota at the phylum level (**A**) and Taxonomic differences between various groups at the phylum level (**B**) of mice with DSS-induced colitis. * *p* < 0.05 and ** *p* < 0.01 indicate a statistically significant difference for challenge (water or DSS). ^#^ *p* < 0.05 and ^##^ *p* < 0.01 indicate a statistically significant difference for dietary treatment (basal or OKG).

**Figure 7:**
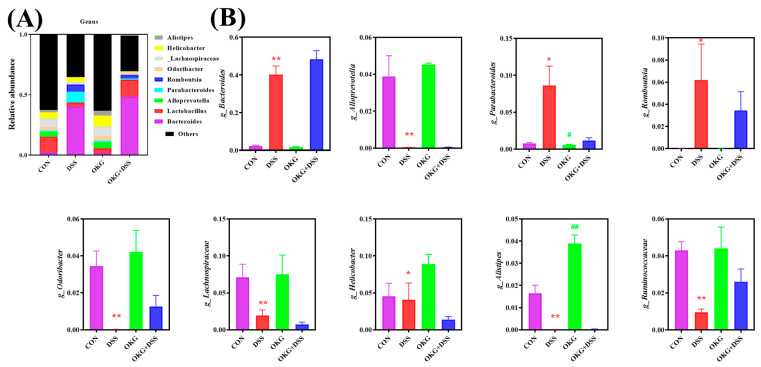
Effects of OKG on the relative abundance of microbiota at the genus level (**A**) and Taxonomy differences between various groups at the genus level (**B**) of mice with DSS-induced colitis. * *p* < 0.05 and ** *p* < 0.01 indicate a statistically significant difference for challenge (water or DSS). ^#^ *p* < 0.05 and ^##^ *p* < 0.01 indicate a statistically significant difference for dietary treatment (basal or OKG).

**Figure 8:**
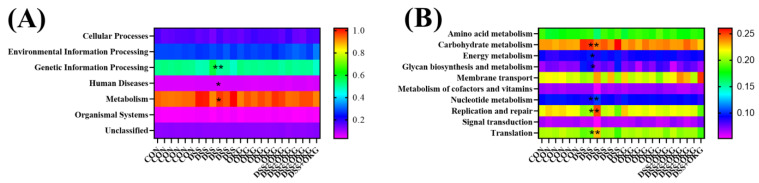
Predictive functional profiling of microbial communities via PICRUSt. KEGG pathway annotations at level 1 (**A**) and level 2 (**B**). * *p* < 0.05 and ** *p* < 0.01 indicate a statistically significant difference for challenge (water or DSS).

Alpha diversity can be characterized by various indices to reflect species richness and evenness in a community. We found that α-diversity was decreased in the DSS mice (Figure 5). At the phylum level, Firmicutes and Bacteroidetes were the dominant phyla in the CON, DSS, OKG, and DSS OKG groups. The proportions of Firmicutes were 34.52%, 31.07%, 46.74%, and 41.53% and those of Bacteroidetes were 60.95%, 55.25%, 45.89%, and 53.96% in the CON, DSS, OKG, and DSS OKG groups, respectively (Figure 6A). In addition, the abundance of Bacteroidetes, Verrucomicrobia, and Melainabacteria decreased markedly, while that of Firmicutes increased markedly in the OKG group (Figure 6B). Compared with the CON group, the levels of Tenericutes and Deferribacteres were significantly decreased, while those of Proteobacteria, Actinobacteria, and Verrucomicrobia were significantly increased in the DSS group. At the genus level, Bacteroides and Lactobacillus were the major genera. The proportions of Bacteroides were 2.23%, 40.19%, 1.67%, and 48.32% and those of Lactobacillus were 13.14%, 3.37%, 4.11%, and 14.18% in the CON, DSS, OKG, and DSS+OKG groups, respectively (Figure 7A). In addition, the levels of Parabacteroides were decreased and the levels of Alistipes were increased in the OKG group (Figure 7B). In the DSS group, the levels of Alloprevotella, Odoribacter, Lachnospiraceae, Helicobacter, Alistipes, and Ruminococcaceae were significantly decreased, while the levels of Bacteroides, Parabacteroides, and Romboutsia were significantly increased. PICRUSt is applied to analyze the functional profiles of microbial communities. By combining 16s sequencing data with genomic databases, it can be used to predict macro-genomic information. The predicted results can be obtained at the KEGG pathway level of second classification (Figure 8). The results showed that the changes in the gut microbiota mainly involve carbohydrate metabolism, membrane transport, replication and repair, and translation. The DSS treatment significantly reduced genetic information processing but increased levels of human diseases and metabolism at level 1. At level 2, the DSS treatment significantly increased carbohydrate metabolism, energy metabolism, and glycan biosynthesis and metabolism but decreased nucleotide metabolism, replication and repair, and translation.

## 4. Discussion

OKG is an amino acid compound salt composed of two molecules of ornithine and one molecule of α-ketoglutarate [30]. It can synthesize glutamine, arginine, proline, and polyamines in vivo [31]. Accumulating evidence has shown that OKG improves nutritional conditions under unhealthy conditions due to its regulatory effect on oxidative stress, tissue injury, and metabolism [12,32,33]. OKG is also more efficient than ornithine and α-ketoglutarate alone [34,35] owing to its extensive hydrogen bond network and electrostatic charges [30]. For example, 0.4 g/kg of OKG increased the content of aspartic acid, proline, alanine, valine, isoleucine, and leucine in fast-growing turkey plasma; increased bone density in tibia trabecular and cortical bone; and improved the mechanical strength of bones [8]. The dietary supplementation of 0.75% OKG significantly increases the daily weight gain and feed intake of nursing piglets [19]. Accordingly, in this study, we found that 1% OKG can increase body weight and serum GH, insulin, and ALP levels and decrease BUN, NH_3_L, and Ile concentrations. These results are similar to reports in previous studies that OKG improved body weight and induced the secretion of anabolic hormones such as insulin and growth hormone [17,35,36]. In vitro, OKG (0.25–2.5 mM) linearly stimulates insulin secretion in rat islets (1.7–4.2 fold) and affects both early- and late-phase insulin secretion kinetics [36]. GH and insulin improve growth, intracellular amino acid transport, protein synthesis, and intestinal absorption [35,37]. In addition, OKG administration increases the catabolism of branched-chain amino acids [38]. ALP is associated with positive growth performance and protein synthesis and reflects the development of bone [39,40]. The decrease in BUN and NH_3_L levels in serum indicates protein synthesis [21]. Thus, 1% OKG may promote growth performance and reduce the concentrations of BUN, NH_3_L, and amino acids in serum by promoting the secretion of GH and the synthesis of protein [41,42]. However, 0.5% or 1.5% OKG treatment tended to increase body weight and average body weight, but the difference was insignificant. These results are similar to reports in previous studies that supplementation with insufficient or excess amounts of amino acids has a negative or no effect on growth performance in animals [22]. Furthermore, 1.5% OKG treatment increases serum UA, NH_3_L, and GLU levels. The non-protein forms of nitrogen mainly include urea and NH_3_L and reflect the balance of amino acids in a diet [43]. Thus, we chose the dosage of 1% OKG to explore the preventive effect of OKG on DSS-induced colitis in mice.

We further discovered that OKG inhibited the pathogenesis of UC by altering the gut microbiome and the secretion of inflammation-related cytokines. Pro-inflammatory cytokines such as TNF-α, IFN-γ, and IL-1 are biomarkers of gastrointestinal diseases [44]. IFN-γ can induce TNF-α production, activate NF-κB signaling pathways, and increase inflammation, making it an important regulator of immune responses [45]. Zhang et al. reported that 2% chlorogenic acid attenuates DSS-induced colitis by reducing DAI, TNF-α levels, histological scores, and colon shortening [46]. Intestinal inflammation can inhibit nutrient absorption, resulting in lower body weights in mice [46]. It is noteworthy that body weight was inhibited in the DSS-induced mice, while OKG alleviated body weight loss, which is similar to OKG’s effect on tumor-bearing rats [47] and pigs induced by D-galactose-associated chronic oxidative stress [17]. In addition, a shortened colon length is a marker and symptom of inflammation [46]. OKG alleviated DSS-induced IBD by improving the DAI score, colon length, and histological changes in mice. Some cytokines damage the intestinal barrier and promote pathogen invasion [48]. We also found that the OKG-treated mice presented significantly increased levels of IL-10, which has been previously confirmed in colon tissue by Sangaraju et al. [49]. IL-10 blocks the activation of NF-κB in order to inhibit the production of other inflammatory cytokines [50]. These observations suggest that OKG exerts a protective effect by suppressing pro-inflammatory markers against DSS-induced colitis.

Gut microbes play a vital role in defense against pathogen invasion [20,51,52,53], and microbial profiles are closely correlated with host immunity and inflammation [46,54]. The disruption of host–gut microbial homeostasis may lead to inflammation [45]. OKG prevents bacterial translocation and spread, thereby reducing enterogenic sepsis [55]. Some studies have reported that DSS-induced mice and UC patients exhibit lower microbial α-diversity [46,56], while 2% chlorogenic acid supplements can improve microbial α-diversity [46]. In this study, we found that DSS reduced microbial α-diversity.

The symptoms of UC normally correspond to higher levels of Bacteroidetes and Proteobacteria and lower levels of Firmicutes in gut microbiota [57]. We further found that OKG enhanced the intestinal abundance of Firmicutes and reduced that of Bacteriodetes, while DSS decreased the intestinal abundance of Firmicutes and increased that of Actinobacteria at the phylum level. These results corroborate the effect of OKG on D-gal pigs and indicate that OKG is closely positively correlated with intestinal microorganisms. The supplementation of 0.5% OKG altered the relative abundance of the gut microbe, especially with respect to Firmicutes and Bacteriodetes, and alleviated body weight loss in chronic oxidative stress models [17]. The abundance of Actinobacteria, Proteobacteria, and Verrucomicrobia was increased in the DSS group, suggesting that these bacteria are highly related to UC [48]. We also observed a decrease in the genera of Ruminococcaceae and Lachnospiraceae and an increase in those of Bacteroides, which have been shown to be involved in enteritis in IBD models [48,54]. In addition, the relative abundance of Parabacteroides was decreased and that of Alistipes was increased in the OKG group. Gentamicin can enhance the abundance of Ruminococcaceae, which repairs the intestinal epithelium [48]. Roman Dziarski also reported that Parabacteroides and Bacteroides enhance, while Alistipes attenuate, colitis in mice [58].

## 5. Conclusions

In summary, dietary supplementation with OKG promotes growth performance and hormone secretion, regulates serum biochemistry and amino acids levels, effectively alleviates growth suppression in DSS-induced mice, represses the release of pro-inflammatory cytokines, and positively impacts the gut microbiota. These findings open a new avenue regarding the mechanisms of action of OKG in nutritional supplementation. However, further research will be necessary to investigate the underlying mechanism by which OKG impacts intestinal barrier function as well as the response of male and female mice to DSS-induced colitis.

## Figures and Tables

**Table 1:** Histological score standards for the colon.

Feature Graded	Description	Grade
Inflammation	None	0
	Slight	1
	Moderate	2
	Severe	3
Extent	None	0
	Mucosa	1
	Submucosa	2
	Transmural	3
Percentage of involvement (%)	1–25	1
	26–50	2
	51–75	3
	76–100	4
Crypt damage	None	0
	Submucosa	1
	Basal one-third lost	2
	Basal two-thirds lost	3
	Only surface epithelium intact	4
	All crypts and epithelia are destroyed	5
Ulceration	No ulceration	0
	Mild ulceration	1
	Moderate ulceration	2
	Extensive ulceration	3

**Table 2:** Effects of OKG on serum biochemical parameters. Data are shown as the mean ± SEM (*n* = 10). Values in the same row with different superscripts represent significantly different findings. The symbol “a, b” indicate that values within a row with different superscripts differ significantly (*p* < 0.05) *.

Item	Control	LOKG	MOKG	HOKG	*p*-Value
TP	27.85 ± 0.90	28.31 ± 0.51	27.84 ± 0.61	29.05 ± 1.12	0.699
ALB	18.45 ± 0.70	18.75 ± 0.37	18.10 ± 0.48	18.73 ± 0.83	0.871
BUN	3.36 ± 0.09 ^a^	2.81 ± 0.18 ^b^	2.77 ± 0.09 ^b^	3.53 ± 0.14 ^a^	0.000
UA	1.17 ± 0.04 ^b^	1.23 ± 0.04 ^ab^	1.30 ± 0.05 ^ab^	1.43 ± 0.09 ^a^	0.048
GLU	1.38 ± 0.10 ^b^	1.55 ± 0.09 ^b^	2.32 ± 0.19 ^a^	2.10 ± 0.13 ^a^	0.000
NH_3_L	273.39 ± 9.44 ^ab^	259.88 ± 9.01 ^ab^	243.01 ± 4.83 ^b^	282.20 ± 16.2 ^a^	0.041
ALT	25.45 ± 1.88 ^a^	25.23 ± 1.25 ^a^	23.73 ± 0.83 ^a^	16.56 ± 1.47 ^b^	0.000
AST	254.40 ± 13.85 ^a^	198.67 ± 14.17 ^ab^	281.40 ± 18.59 ^a^	64.80 ± 8.91 ^b^	0.000
ALP	48.50 ± 3.01 ^b^	59.60 ± 3.67 ^a^	64.63 ± 2.85 ^a^	54.00 ± 0.26 ^ab^	0.002

* TP (Total Protein); ALB (Albumin); BUN (Blood urea nitrogen); UA (Uric Acid); GLU (Glucose); NH_3_L (Blood ammonia); ALT (Alanine transaminase); AST (Glutamic-oxalacetic transaminase); ALP (alkaline phosphatase).

**Table 3:** Effects of OKG on serum amino acids. Data are expressed as the mean ±SEM (*n* = 10). Values in the same row with different superscripts are significantly different. The symbol “a, b” indicates that values within a row with different superscripts differ significantly (*p* < 0.05) *.

Item	Control	LOKG	MOKG	HOKG	*p*-Value
Asp	7.21 ± 0.27	7.15 ± 0.18	7.47 ± 0.22	7.62 ± 0.30	0.509
Thr	21.02 ± 1.33	20.78 ± 0.39	21.27 ± 0.44	20.22 ± 1.04	0.874
Ser	9.54 ± 0.46	9.19 ± 0.24	8.92 ± 0.41	9.48 ± 0.63	0.758
Glu	20.10 ± 0.88	19.02 ± 0.48	18.88 ± 0.67	21.81 ± 1.64	0.159
Gly	8.94 ± 0.27	9.34 ± 0.29	9.33 ± 0.21	8.88 ± 0.48	0.663
Ala	15.29 ± 1.17	13.74 ± 0.49	14.15 ± 0.81	13.70 ± 0.43	0.470
Cys	12.17 ± 0.70 ^b^	12.66 ± 0.37 ^b^	13.23 ± 0.20 ^b^	16.84 ± 0.37 ^a^	0.000
Val	9.87 ± 0.95	8.09 ± 0.35	8.08 ± 0.05	9.56 ± 1.12	0.271
Met	9.52 ± 0.79	8.04 ± 0.22	9.23 ± 0.35	9.63 ± 0.45	0.159
Ile	3.36 ± 0.32 ^a^	2.37 ± 0.05 ^b^	2.39 ± 0.12 ^b^	2.96 ± 0.31 ^ab^	0.028
Leu	6.27 ± 0.46	5.25 ± 0.12	7.88 ± 1.86	6.86 ± 1.27	0.427
Tyr	4.03 ± 0.67 ^ab^	2.89 ± 0.26 ^b^	7.28 ± 1.71 ^a^	4.42 ± 1.48 ^ab^	0.039
Phe	3.41 ± 0.16 ^b^	4.39 ± 0.32 ^b^	7.31 ± 0.62 ^a^	6.84 ± 0.63 ^a^	0.000
Lys	25.53 ± 1.43	26.72 ± 0.81	27.03 ± 1.84	26.76 ± 1.42	0.889
His	4.59 ± 0.76 ^b^	4.86 ± 0.66 ^b^	6.15 ± 0.99 ^ab^	7.70 ± 1.17 ^a^	0.046
Arg	10.82 ± 0.74	10.42 ± 0.38	10.40 ± 0.37	11.73 ± 0.70	0.365
Pro	12.69 ± 0.67	10.54 ± 0.45	12.17 ± 0.90	10.53 ± 0.86	0.095

* Asp (Aspartic acid); Thr (Threonine); Ser (serine); Glu (Glutamic acid); Gly (Glycine); Ala (Alanine); Cys (Cysteine); Val (Valine); Met (Methionine); Ile (Isoleucine); Leu (Leucine); Tyr (Tyrosine); Phe (Phenylalanine); Lys (Lysine); His (Histidine).

## Data Availability

Not applicable.
